# MPOWER Tobacco Control Policies’ Effects on Lip and Oral Cavity Cancer Trends in MERCOSUR Countries

**DOI:** 10.3390/ijerph22040644

**Published:** 2025-04-19

**Authors:** Laila Menezes Hagen, Fernanda Joly Macedo, Amanda Ramos da Cunha, Fernando Neves Hugo, José Miguel Amenábar

**Affiliations:** 1 Department of Stomatology, Federal University of Paraná, Curitiba 80210-170, PR, Brazil; lailahagen@ufpr.br (L.M.H.); fernandajoly@ufpr.br (F.J.M.); 2 Department of Epidemiology, School of Public Health, University of São Paulo, São Paulo 01246-904, SP, Brazil; amandaracunha@usp.br; 3 Department of Epidemiology and Health Promotion, New York University College of Dentistry, New York, NY 10010, USA; fnh9064@nyu.edu

**Keywords:** oral cancer, tobacco use, tobacco control, MERCOSUR

## Abstract

Background: As tobacco is the main risk factor for oral cancer, it is important to understand the burden of this disease in light of the Framework Convention on Tobacco Control. For MERCOSUR, tobacco control is a topic of interest. The aim of this study was to describe MPOWER measure scores and to evaluate the trends in lip and oral cavity cancer (LOC) incidence, mortality and Disability-Adjusted Life Years (DALYs), from 2005 to 2021, in MERCOSUR countries. Methods: This is an ecological descriptive study, where the MPOWER measure scores were extracted from the Global Health Observatory of World Health Organization, and the Age-standardized Rates (ASRs) of LOC for MERCOSUR countries were obtained from the Global Burden of Diseases 2021 results tool. The trends of LOC ASRs were obtained using the Prais–Winsten method. Results: Paraguay and Venezuela had lower MPOWER scores over time than Argentina, Brazil and Uruguay. These last three countries showed LOC trends decreasing for most indicators among males, while Paraguay and Venezuela showed increasing trends in incidence for both males and females, and no decreasing trends. Conclusion: This study suggests that MERCOSUR countries with a history of more rigorous MPOWER tobacco control policies are exhibiting decreasing trends in LOC burden.

## 1. Introduction

Oral cancer is a disease characterized by high mortality, with an overall survival rate of approximately 54% [1], and a significant impact on survivors’ quality of life, primarily due to chronic pain, functional impairments affecting speech and swallowing, and psychological distress [2]. As a result, oral cancer imposes a substantial global burden [3]. In 2019, lip and oral cavity cancer (LOC) was the 13th leading cause of cancer-related Disability-Adjusted Life Years (DALYs) globally, with an age-standardized DALY rate of 66.1 per 100,000 inhabitants [4]. More recently, in 2021, the DALY rate for LOC was found to be even higher, reaching 74.44 per 100,000 [5]. Furthermore, between 1990 and 2021, the incidence, mortality, and DALY rates of LOC have increased globally, with the most significant rise observed in countries with a mid-level Sociodemographic Index (SDI) [5].

With a higher prevalence in men from the fifth decade of life, squamous cell carcinoma is the most common histological type of oral cancer, and the primary etiological factors are alcohol and tobacco, which exert a synergistic effect on the development of this cancer [6,7]. Due to its carcinogenic compounds, tobacco exhibits a dose–response relationship between the number of packs smoked per year and the risk of developing oral cancer [8]. Moreover, smoking is not the only form of tobacco use—chewing tobacco is a common habit, particularly in Southeast Asia, which contributes to the high burden of oral cancer in this region [3,8].

To address and attempt to reduce the extreme harm caused by the global tobacco epidemic, the World Health Organization launched, in 2003, the Framework Convention on Tobacco Control (WHO-FCTC), which entered into force in 2005 [9]. In 2008, MPOWER, a technical package of measures, was introduced to help countries to reduce the demand for tobacco, which encompasses six measures: (M) monitoring tobacco use and prevention policies; (P) protecting people from tobacco smoke; (O) offering help to quit tobacco use; (W) warning about the dangers of tobacco; (E) enforcing bans on tobacco advertising, promotion and sponsorship; and (R) raising taxes on tobacco [10].

High rates of DALYs of cancer attributed to behavior risk factors are seen in some Latin American countries [11]. However, smoking-related LOC deaths are declining in America as a whole [12]. In the context of the MERCOSUR (Southern Common Market), composed of Argentina, Brazil, Paraguay, Uruguay and Venezuela, tobacco control has been one of the topics of interest since 2003, when the Intergovernmental Commission for Tobacco Control in MERCOSUR was created, which was important for identifying and planning priorities for tobacco control by the MERCOSUR member countries [13]. Although these five countries have already adhered to the various strategies recommended by the WHO, there are regional challenges, such as socioeconomic and cultural diversity, making it difficult to fully achieve the MPOWER goals [14,15].

More than 20 years since the WHO-FCTC was launched, it is important to investigate the burden of non-communicable diseases in recent years. The aim of this study was to describe MPOWER measure scores and to evaluate the trends of LOC incidence, mortality and DALYs, from 2005 (year of WHO-FCTC implementation) to 2021, in MERCOSUR countries.

## 2. Materials and Methods

This is an ecological descriptive study, where the scores of the MPOWER measures and, using a time series analysis, the trends of the LOC Age-standardized Rates (ASRs) were described. The units of analysis were the five countries that are part of MERCOSUR.

### 2.1. MPOWER Scores Data Source

The MPOWER scores were extracted from the Global Health Observatory of the WHO [16]. The MPOWER scores started to be attributed in 2007, except for the R measure, which started in 2008. Since 2008, the scores have been attributed biennially. In this study, the MPOWER scores of 2007, 2008, 2010, 2012, 2014, 2016, 2018 and 2020 were included [10]. MPOWER scores are based on official country reports, surveys collected by WHO Regional and WHO Country Offices, and original national tobacco control legislation. The data are analyzed by experts, as detailed in the Technical Note of the WHO Report on the Global Tobacco Epidemic [17]. The M score is the only one that varies between 1 and 4, while POWER measures vary between 1 and 5. A score of 1 always means that there was not enough information about the measure in the country. As the score goes up, the quality or intensity of the measure is better [10].

### 2.2. Lip and Oral Cavity Cancer Rates Data Source

LOC ASRs from 2005 to 2021 of MERCOSUR countries were obtained from the Global Burden of Diseases (GBD) 2021 study results tool (Global Health Data Exchange—GHDx) [18]. The GBD uses sophisticated methods to estimate the distribution of diseases and injuries in 204 countries and territories, with many collaborators from around the world [3]. The process of LOC estimates was explained in detail by GBD collaborators in a recent paper [3]. For this study, ASRs were selected, and the metrics included were incidence, mortality, and DALYs. Sexes were selected separately, since they have historically presented distinct characteristics for LOC [7]. Sites included in the LOC are represented by codes C00-C08 of the International Statistical Classification of Diseases and Related Health Problems, Tenth Revision (ICD-10) [3].

### 2.3. Statistical Analysis

MPOWER measure scores were descriptively analyzed by extracting the medians and interquartile range for each measure and for the sum of all the measures. The trends of LOC ASRs were analyzed using the Prais–Winsten generalized linear regression method, which accounts for first-order autocorrelation in time series data [19]. Subsequently, the Annual Percent Change (APC) and its 95% confidence interval (95% CI) were calculated following the methodology described by Antunes and Waldman [19]:

APC = (−1 + 10^β^_1_) × 100

To estimate the 95% CI, the confidence bounds of β_1_*^lower^* and β_1_*^upper^* were calculated as follows:

β_1_*^lower^* = −t_n−2_ × SE(β_1_)

β_1_*^upper^* = β_1_ + t_n−2_ × SE(β_1_)

where t_n−2_ is the critical value of the t-distribution with n−2 degrees of freedom (α = 0.05), and SE(β_1_) is the standard error of the coefficient β_1_). These confidence bounds were then transformed to the APC scale using base 10, applying the following formulas:

APC lower = (−1 + 10^β^_1_*^lower^*) × 100

APC upper = (−1 + 10^β^_1_*^upper^*) × 100

β_1_*^upper^* and β_1_*^lower^* represent the limits of the confidence interval, respectively. The APC and its 95% CI are represented by the trend, which can be (1) decreasing, when the APC and the 95% CI are negative; (2) stationary, when the lower value of 95% CI is negative and the higher is positive (including zero in the interval); (3) increasing, when the APC and the 95% CI are positive. The analysis was conducted using the Statistical Package for the Social Sciences (SPSS) software, version 20, and Stata Software, version 13.1.

## 3. Results

### 3.1. MPOWER Scores

**Figure 1:**
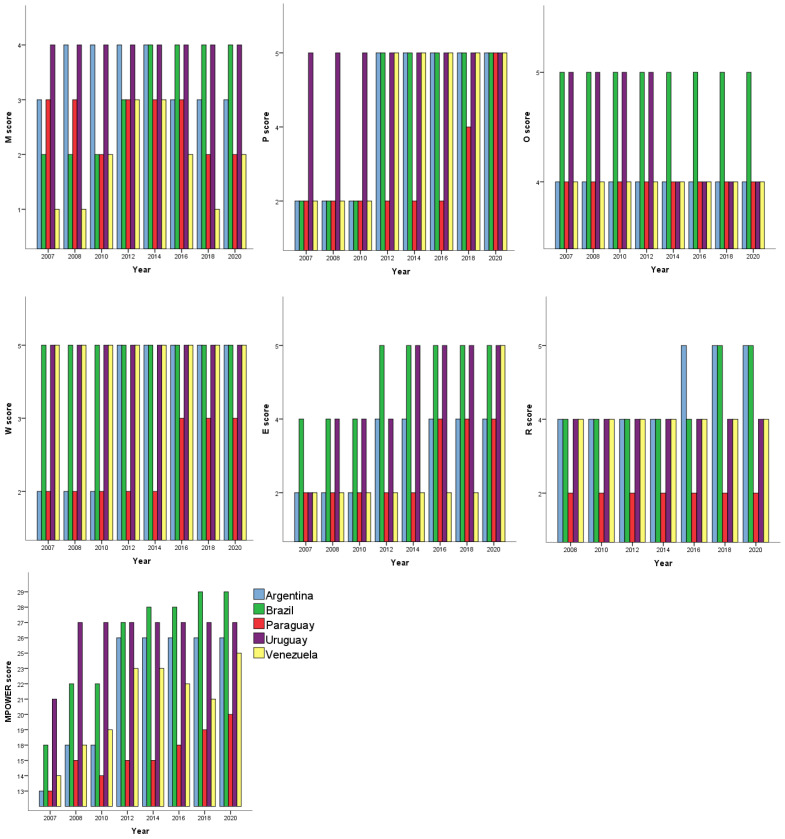
MPOWER measure scores of MERCOSUR countries, in 2007, and then biennially, between 2008 and 2020.

Figure 1 shows the distribution of scores over time for each MPOWER measure, as well as all of them together. Paraguay scores were low mainly for W and R measures when compared to the other MERCOSUR countries over time. Venezuela scores were low for M and E measures. Argentina started with low scores in P, W and E measures, but in 2012 the scores for these measures rose. Table 1 shows these results summarized in medians and interquartile ranges for each MPOWER measure and for their sum. Paraguay exhibited the lower medians, followed by Venezuela. Brazil and Uruguay had the highest medians, while Argentina was not far behind.

### 3.2. LOC Trends

Table 2 reveals the LOC trends for males and females in each country. Among males, Argentina, Brazil and Uruguay had decreasing trends for almost all measures; the exception was incidence in Uruguay, which was stationary. Argentina showed the largest decreasing APCs among countries. Among females, Argentina showed all measures as stationary, Brazil showed incidence as stationary and mortality and DALYs decreasing, and Uruguay showed increasing incidence, stationary mortality and decreasing DALYs. On the other hand, Paraguay and Venezuela had no decreasing trends for males or females; both had increasing incidence trends for both sexes, while the other measures were stationary.

**Figure 2:**
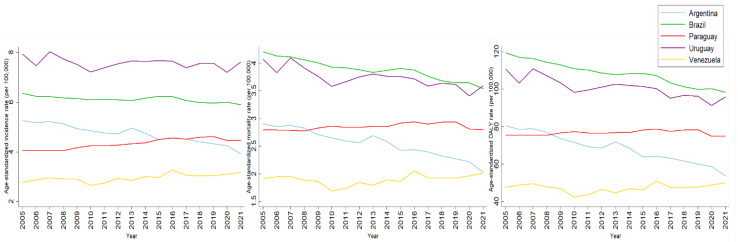
Incidence, mortality and DALY age-standardized rates of lip and oral cavity cancer among males in MERCOSUR countries between 2005 and 2021.

Figure 2 shows the paths of LOC ASRs for males, between 2005 and 2021. Uruguay had the highest incidence of ASRs throughout the period. However, mortality and DALY ASRs of Brazil were higher than those of Uruguay. Despite this, mortality and DALY rates in Brazil have been decreasing notably. ASRs of all indicators for males in Argentina were also notably decreasing, and, on the other hand, Paraguay, which began the period analyzed with lower ASRs than Argentina, surpassed its ASRs in all indicators. Venezuela’s ASRs for all indicators for males fluctuated, but in the last decade they were increasing.

**Figure 3:**
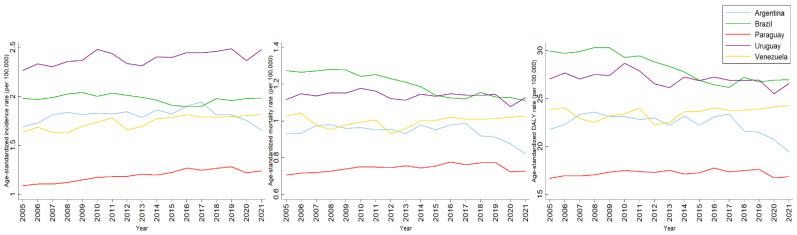
Incidence, mortality and DALY age-standardized rates of lip and oral cavity cancer among females in MERCOSUR countries between 2005 and 2021.

Figure 3 shows the paths of LOC ASRs for females, between 2005 and 2021. The level of ASRs was lower when compared with males. Similarly to the scenario for males, the incidence of ASRs for Brazil were lower than in Uruguay, but mortality and DALYs were higher. Despite this, in recent years, these ASRs have been decreasing for females in Brazil, while in Uruguay they are fluctuating. Only in the last few years of the time series have Argentina ASRs been decreasing. Also similar to males, Venezuela’s ASRs fluctuated over time, but in the last decade they seemed to be increasing. Paraguay had the lowest ASRs for all indicators over the entire period, but they appeared to be slowly and steadily increasing.

## 4. Discussion

In this study, it was observed that Paraguay and Venezuela are the MERCOSUR countries with the lowest MPOWER scores and the only countries that have not shown any indicator of LOC with a decreasing trend between 2005 and 2021. On the other hand, Argentina, Brazil and Uruguay showed progression in MPOWER scores and reductions in LOC ASRs, especially for men. For these three countries with the best MPOWER scores, the only increasing indicator was incidence for women of Uruguay; all the other trends were decreasing or stationary.

The impact of the WHO-FCTC on the burden of tobacco-related diseases remains unclear. However, studies suggest that the strong implementation of MPOWER measures could significantly reduce tobacco-related mortality in the future [20] and, more specifically, prevent numerous cancer cases [21]. A study by Ramadan et al. [21] examined the relationship between the implementation of MPOWER tobacco control measures and lung cancer incidence in Saudi Arabia. Their findings indicate that as MPOWER scores improved nationwide, lung cancer incidence gradually declined.

In Latin American countries, lung cancer has exhibited varying trends recently. A study analyzing cancer mortality trends in South American countries from 1990 to 2020 or 2000 to 2020 found that, while Venezuela and Paraguay were among the few countries that did not exhibit declining trends in male cancer mortality across certain age groups, Brazil was also included among these countries, showing increasing trends in lung cancer mortality for males across all age groups. For females, most countries displayed increasing mortality trends, with Paraguay being an exception, showing a stable mortality trend. For the Brazil analysis, however, the period was large, comprising 1990 to 2020, and it is possible to see that after 2000 some trends were starting to decline [22]. Another study assessed the trends in mortality and DALYs for tracheal, bronchial, and lung cancer attributable to smoking in South America and found different results. While most countries showed declining trends in mortality and DALYs among males, Paraguay exhibited increasing trends when comparing the periods of 2015–2019 and 1990–1994. However, among females, Argentina and Uruguay saw the highest increases in both mortality and DALYs [23].

To our knowledge, the first study to assess the global impact of the WHO-FCTC on oral cancer [24] used an interrupted time series analysis, revealing that countries with higher MPOWER scores exhibited declining trends in oral cancer rates following the adoption of the WHO-FCTC, compared to the trends observed in the preceding period. Therefore, continuous monitoring of cancer trends is crucial to determine whether tobacco control measures are starting to yield results or if alternative strategies need to be explored.

Socioeconomic, cultural, and political factors influence how tobacco control policies impact tobacco use prevalence [25]. Additionally, socioeconomic development also influences the distribution of the oral cancer burden. In Latin American countries where MPOWER measures have been less effectively implemented, smoking has shown a stronger association with oral cancer mortality [14]. Recent time series analyses have also revealed varying trends in oral cancer incidence across MERCOSUR countries, depending on the specific period analyzed [14,15,26].

Herrera-Serna et al. [14] reported that Paraguay had made limited advances in tobacco and alcohol control policies over the years. In their study, the period under analysis was 2000 to 2017, and Paraguay demonstrated stationary trends for oral cancer mortality for both men and women. Venezuela also did not progress ideally in some tobacco and alcohol control measures, and, between 2000 and 2017, oral cancer mortality rates were stationary for men; however, they decreased for women. In our study, additional information was shown about incidence trends, which were increasing for men and women in Paraguay and Venezuela. The oral cancer scenario shown in Paraguay and Venezuela could be related to the slow process of these countries implementing the tobacco control policies, reinforcing the need to intensify these measures. As seen in this study, Paraguay had, and still has, a low score on the ‘raise taxes on tobacco’ measure, which is one of the most effective measures for tobacco control [25,27], and needs to be addressed. Venezuela must particularly improve its tobacco use monitoring and tobacco control policies, which have worsened in recent years.

In this study, Argentina, Brazil and Uruguay presented better progress in the MPOWER measures. Most of the trends among men for these countries were decreasing. Brazil has reduced the prevalence of tobacco use greatly in recent decades [14,28,29]. In this study, Brazil was the country with more decreasing indicators of LOC, with almost all trends decreasing, except for incidence in women, which was stationary. However, Argentina’s APCs among men showed greater declines than in Brazil, suggesting that LOC reductions in Brazil, especially for incidence rates, do not appear to be reducing proportionately to what might be expected based on the large reduction in tobacco prevalence. Brazil is a large country, with many particularities. In addition to the differences in tobacco control that could be happening over the country, other risk factors could be influencing the oral cancer burden, such as alcohol and, very importantly, socioeconomic factors [30,31].

In South America, Brazil and Uruguay have one of the highest rates of mortality of oral cancer [3] and, despite the decreasing ASRs of mortality and DALYs for men in Brazil, there was a discrepancy when compared with Uruguay. Brazil had lower incidence rates than Uruguay, but higher mortality and DALYs ASRs. It means that LOC in Brazil proportionally kills and debilitates more people than in Uruguay. It is possible that socioeconomic inequalities in Brazil are, once again, playing an important role in these results, since these factors are associated with higher oral cancer mortality rates in Latin American countries [32] and Brazil has one of the highest Gini indices in the region [15]. This topic is very important in reinforcing how socioeconomic inequalities influence health outcomes and need to be addressed.

Uruguay has had high scores for MPOWER measures since when the scores started to be attributed. According to Abascal et al. [33], between 2005 and 2011, Uruguay reduced its tobacco consumption per person by 4,3% annually. However, only mortality and DALY ASRs in men have been decreasing, while incidence ASRs in women have been increasing in recent years. These results converge with Herrera-Serna et al. [26], which, between 2000 and 2020, also found this decrease for men, while for women there were increasing trends. These findings need to be further explored and understood. Abascal et al. [33] found that women students from Uruguay, between 2001 and 2009, had higher tobacco use prevalence than male students, with a female:male ratio of 1.32, which could be an important research topic for future research in Uruguay.

Although it is important to note that Argentina is one of the few Latin American countries that has not ratified the WHO-FCTC, having only signed it [10], after the initial years, Argentina was not far behind Brazil and Uruguay in MPOWER scores. However, tobacco use was previously decreasing in Argentina [34], which could be related with the higher decreases observed in LOC trends for all men indicators in this study. Furthermore, the trends for women were stationary, but it was possible to observe that in the last few years of the period the rates were in decline. Considering these results, Argentina’s scenario for oral cancer may be promising for the next decades.

The illegal trade of tobacco is an important topic that must be addressed in this context and is a priority for the Intergovernmental Commission for Tobacco Control in MERCOSUR [12]. WHO-FCTC also addressed this problem, creating a protocol to eliminate illicit trade in tobacco products [35]. For MERCOSUR countries, it is an important problem, mainly because of the illegal trade of low-price and not-known brands, which can seem a better option for people with a low socioeconomic status [36]. This illicit trade influences how the tobacco control measures will affect the prevalence of tobacco in a population, since it increases the access to tobacco, being cheaper [35]. Paraguay is the main supplier of illegal cigarettes in Latin America [37], which reinforces the need for stronger policies in this country [36].

### Limitations

Studies carried out with secondary data are subject to a series of limitations, such as data sources being of differing quality and reliability. However, GBD methodologies are sophisticated, and every year are improving, resulting in representative estimates [38]. Another limitation of this study is that oral cancer has other risk factors that influence the distribution of the disease, such as alcohol, diet and socioeconomic level [39]. For example, in 2019, the proportion of LOC DALYs attributed to alcohol consumption in MERCOSUR countries ranged from 36% in Venezuela to 53% in Argentina [3]. Additionally, a low intake of fruits and vegetables increases the risk of oral cancer [40], and low socioeconomic status—including low income, limited education, and lower occupational social class [30]—also plays a significant role, although the proportion of oral cancer cases attributable to these factors remains unknown. Moreover, a fraction of oral cancer cases occur without any known risk factors, prompting researchers to explore alternative pathways for the disease [41].

The descriptive analysis of this study is another known limitation; however, we believe that this is the best way to evaluate in detail the scenario of the five MERCOSUR countries in an exploratory way. MPOWER scores are reliable data to assess the strength of tobacco control measures in countries that have adhered to the WHO-FCTC and also represent the effort that the country has been making towards tobacco control. Although the time passed might still be too short to confirm the effects of WHO-FCTC in oral cancer rates, it is imperative to begin to understand the different scenarios and needs of countries, especially in Latin American, where there are countries with complex socioeconomic aspects that need to be addressed.

## 5. Conclusions

This study suggests that MERCOSUR countries with a history of more rigorous MPOWER tobacco control policies are exhibiting decreasing trends in LOC burden, mainly among men. In contrast, Paraguay and Venezuela, which had the lowest MPOWER scores, did not show similar declines, highlighting the need to strengthen their tobacco control measures.

## Figures and Tables

**Table 1:** Median and interquartile range (IQR) of MPOWER scores of MERCOSUR countries.

Countries	M	P	O	W	E	R	MPOWER
Argentina	3.50 [3; 4]	5.0 [2; 5]	4.0 [4; 4]	5.0 [2; 5]	4.0 [2; 4]	4.5 [4; 5]	26.0 [18; 26]
Brazil	3.50 [2; 4]	5.0 [2; 5]	5.0 [5; 5]	5.0 [5; 5]	5.0 [4; 5]	4.0 [4; 5]	27.5 [22; 28.3]
Paraguay	3.0 [2; 3]	2 [2; 2.5]	4.0 [4; 4]	2.0 [2; 3]	2 [2; 5]	2.0 [2; 2]	15.0 [14.8; 19.3]
Uruguay	4.0 [4; 4]	5.0 [5; 5]	4.5 [4; 5]	5.0 [5; 5]	4.5 [4; 5]	4.0 [4; 4]	27.0 [27; 27]
Venezuela	2.0 [1; 2.25]	5.0 [2; 5]	4.0 [4; 4]	5.0 [5; 5]	2 [2; 2]	4.0 [4; 4]	21.5 [8.8; 23]

**Table 2:** Incidence, mortality and DALY ASR trends: Annual Percent Change (APC) and 95% Confidence Intervals (95% CI) of time series (2005 to 2021).

Country	Sex	Incidence Trend (APC [95% CI])	Mortality Trend (APC [95% CI])	DALYs Trend (APC [95% CI])
Argentina	Male	Decreasing (−1.55 [1.87; −1.24])	Decreasing (−1.98 [−2.39; −1.56])	Decreasing (−2.24 [−2.56; −1.92])
	Female	Stationary (−0.02 [−0.76; 0.74])	Stationary (−0.65 [−1.50; 0.21])	Stationary (−0.63 [−1.40; 0.14]
Brazil	Male	Decreasing (−0.37 [−0.59; −0.15])	Decreasing (−0.98 [−1.20; −0.76])	Decreasing (−1.16 [−1.34; −0.98])
	Female	Stationary (−0.10 [−0.46; 0.27])	Decreasing (−0.95 [−1.27; −0.63])	Decreasing (−0.79 [−1.22; −0.36])
Paraguay	Male	Increasing (0.74 [0.39; 1.09])	Stationary (0.14 [−0.18; 0.46])	Stationary (0.05 [−0.22; 0.33])
	Female	Increasing (0.91 [0.58; 1.24])	Stationary (0.27 [−0.16; 0.71])	Stationary (0.08 [−0.18; 0.34])
Uruguay	Male	Stationary (−0.21 [−0.50; 0.07])	Decreasing (−0.72 [−1.06; −0.38])	Decreasing (−0.89 [−1.18; −0.59])
	Female	Increasing (0.40 [0.14; 0.66])	Stationary (−0.13 [−0.34; 0.09])	Decreasing (−0.27 [−0.53; −0.02])
Venezuela	Male	Increasing (0.77 [0.26; 1.27])	Stationary (0.31 [−0.43; 1.07])	Stationary (0.24 [−0.47; 0.96])
	Female	Increasing (0.67 [0.42; 0.93])	Stationary (0.12 [−0.32; 0.56])	Stationary (0.21 [−0.12; 0.54])

## Data Availability

The data used in this study are estimates resulting from the 2021 GBD study. They are available on the website of the Institute for Health Metrics and Evaluation [18], and can be publicly consulted and downloaded.
